# Fatiguing high-intensity intermittent exercise depresses maximal Na^+^-K^+^-ATPase activity in human skeletal muscle assessed using a novel NADH-coupled assay

**DOI:** 10.1007/s00424-024-03036-6

**Published:** 2024-11-14

**Authors:** Jeppe F. Vigh-Larsen, Sara M. Frangos, Kristian Overgaard, Graham P. Holloway, Magni Mohr

**Affiliations:** 1 Department of Public Health, Research Unit in Exercise Biology, Aarhus University, Aarhus, Denmark; 2 Department of Sports Science and Clinical Biomechanics, University of Southern Denmark, Odense, Denmark; 3 Department of Human Health and Nutritional Sciences, University of Guelph, Guelph, ON Canada; 4 Centre of Health Science, University of the Faroe Islands, Tórshavn, Faroe Islands

**Keywords:** Excitability, Excitation–contraction coupling, Potassium, Fatigue, Glycogen, Carbohydrate

## Abstract

The Na^+^-K^+^-ATPase is a critical regulator of ion homeostasis during contraction, buffering interstitial K^+^ accumulation, which is linked to muscle fatigue during intense exercise. Within this context, we adopted a recently reported methodology to examine exercise-induced alterations in maximal Na^+^-K^+^-ATPase activity. Eighteen trained healthy young males completed a repeated high-intensity cycling protocol consisting of three periods (EX1-EX3) of intermittent exercise. Each period comprised 10 × 45-s cycling at ~ 105% W_max_ and a repeated sprint test. Muscle biopsies were sampled at baseline and after EX3 for determination of maximal in vitro Na^+^-K^+^-ATPase activity. Blood was drawn after each period and in association with a 2-min cycling test at a standardized high intensity (~ 90% W_max_) performed before and after the session to assess plasma K^+^ accumulation. Further, a 5-h recovery period with the ingestion of carbohydrate or placebo supplementation was implemented to explore potential effects of carbohydrate availability before sampling a final biopsy and repeating all tests. A ~ 12% reduction in maximal Na^+^-K^+^-ATPase activity was demonstrated following EX3 compared to baseline (25.2 ± 3.9 vs. 22.4 ± 4.8 μmol·min^−1^·g^−1^ protein, *P *= 0.039), which was sustained at the recovery time point (~ 15% decrease compared to baseline to 21.6 ± 5.9 μmol·min^−1^·g^−1^ protein, *P* = 0.008). No significant effect of carbohydrate supplementation was observed on maximal Na^+^-K^+^-ATPase activity after recovery (*P* = 0.078). In conclusion, we demonstrate an exercise-induced depression of maximal Na^+^-K^+^-ATPase activity following high-intensity intermittent exercise, which was sustained during a 5-h recovery period and unrelated to carbohydrate availability under the present experimental conditions. This was shown using a novel NADH coupled assay and confirms previous findings using other methodological approaches.

## Introduction

Interstitial K^+^ accumulation has been proposed to play a central role in muscle fatigue during intense contractile activity [1–4]. Indeed, K^+^ accumulation may lead to depolarization of the resting membrane potential, and if severe, this depolarization may induce muscle fiber inexcitability due to slow-inactivation of voltage-gated Na^+^ channels [5]. In humans during exercise, interstitial K^+^ concentrations of 10–12 mM at the point of fatigue have been reported [6, 7], which is sufficient to cause a depolarization that reduces muscle force production based on in vitro scenarios [8].

In addition to the excitation-induced passive fluxes of K^+^ and Na^+^, the regulation of muscle ion homeostasis is governed by an intricate system of membrane proteins including muscle chloride channels (ClC-1), ATP-dependent K^+^ channels (K_ATP_) and the sodium–potassium pump (Na^+^-K^+^-ATPase) as recently reviewed in detail [8]. A rapid contraction-induced activation of the Na^+^-K^+^-ATPase constitutes one of the primary protective mechanisms to counter K^+^-induced force depression [9–11]. Indeed, high-frequency muscle activation can elicit a ~ 20-fold increase in Na^+^-K^+^-ATPase activity within 10 s, approaching the theoretical maximal capacity using all available pumps [12, 13]. The key role of this activation is shown by significantly accelerated force depression when reducing Na^+^-K^+^-ATPase capacity with ouabain [14] or with inadequate glycolytic ATP production to sustain maximal enzyme activity [15]. Moreover, it is a common finding that Na^+^-K^+^-ATPase subunits are upregulated in response to exercise training [3] and associated with improved interstitial K^+^ regulation and high-intensity exercise performance [7, 16].

In contrast to the contraction-induced activation of the Na^+^-K^+^-ATPase, depression of maximal pump capacity has been shown after intense exercise in humans, which may render muscle cells prone to K^+^-induced fatigue progression [17]. Indeed, decreases in maximal pump activity of ~ 10–20% (ranging from ~ 5–44%) have been demonstrated following various endurance or resistance-type activities [18–30]. Importantly, these reductions occurred without alterations in Na^+^-K^+^-ATPase content and were attributed to potential metabolic and/or structural perturbations of the enzyme [26]. For example, muscle glycogen availability [15, 31–33] or post-translational protein modifications (e.g. glutathionylation) have been proposed to modulate maximal pump capacity [34–36].

Notably, exercise-induced reductions in maximal Na^+^-K^+^-ATPase activity are not a consistent finding. No change, or even increases in maximal Na^+^-K^+^-ATPase activity [37–39] have been reported in humans after exhaustive cycling [40], in electrically stimulated isolated rat soleus muscle in vitro [41] and following treadmill running in rats [42], underscoring the ambiguity of this response. In addition to the specific exercise modalities adopted, these discrepant findings may relate to the different methods applied to measure maximal Na^+^-K^+^-ATPase activity. In humans, two main assays in crude muscle homogenate have been employed, i) the maximal K^+^-stimulated 3-O-MFPase assay [43, 44] and ii) direct measurements of K^+^ or Na^+^ dependent ATP hydrolysis rates measured through ^33^P-ATP [42]. While the former has been criticised for not being Na^+^-dependent and only detecting one component of the Na^+^-K^+^-ATPase cycle (phosphatase activity), the latter is sensitive to background contamination from other ATPases and has a very low activity in purified membrane fractions [3]. Moreover, contradictory findings have been reported comparing these approaches [45], highlighting a complexity, which may reflect methodological as well as biological aspects.

Interestingly, a new method was recently developed to determine Na^+^-K^+^-ATPase activity using an NADH coupled assay with the addition of a myosin-ATPase inhibitor to limit background noise and enable reliable measurements of Na^+^-K^+^-ATPase activity [46]. Notably, this assay yielded three times higher maximal rates than previously published results [25, 42], approaching the theoretical maximal limit [47]. The primary aim of the present study was therefore to apply this assay to assess potential changes in maximal Na^+^-K^+^-ATPase activity in response to a high-intensity intermittent exercise regimen. As a secondary and more explorative aspect, the response to subsequent carbohydrate supplementation during a 5-h recovery period to manipulate muscle glycogen stores was also investigated. We hypothesized that the intense exercise modality would reduce maximal Na^+^-K^+^-ATPase activity and that subsequent carbohydrate supplementation would potentially accelerate the recovery of enzyme function.

## Materials and methods

This study is part of a larger data collection with additional manuscripts published presenting other parts of the investigation [48–50]. Approval was obtained by the Central Denmark Region Committees on Health Research (application number 1–10-72–15-20) and the project conformed to the standards of the Declaration of Helsinki, except for database registration. All participants were informed of potential risks and discomforts associated with study participation and written informed consent was obtained.

### Participants

Twenty healthy young males were recruited for the study with two subsequently excluded during the experimental days because of technical problems with a cycle ergometer. Thus, data from 18 participants are included in the manuscript (age: (mean ± SD) 25 ± 2 years; body mass: 78 ± 9 kg; body fat: 9.4 ± 2.5%; and VO_2max_: 57 ± 5 ml·kg^−1^·min^−1^). The myosin heavy chain (MHC) distribution of the participants was 51 ± 6% type 1 fibers, 45 ± 5% type 2a fibers and 5 ± 4% type 2 × fibers. Inclusion criteria were a VO_2max_ above 50 ml·kg^−1^·min^−1^ and familiarity with cycling exercise, while exclusion criteria were any injuries or illnesses limiting participation. After completing a main session of high-intensity intermittent exercise, the participants were randomized into two groups for a 5-h recovery period (placebo or carbohydrate supplementation; *n* = 9 each) with no significant group differences in the participant characteristics, as reported previously [49], except a small difference in age (24.0 ± 1.3 vs. 26.4 ± 2.4 years, *P* = 0.015 in the carbohydrate vs. placebo-supplemented group). The sample size was based on a different primary outcome presented in a previous paper [49]. However, to ensure adequate power for the present analyses, we also performed a separate sample size calculation revealing that a minimum of 8 participants would be needed to detect a 10% decline in Na^+^-K^+^-ATPase activity with an SD of 10%. Given uncertainty around the magnitude of variation in post-exercise changes with the use of this novel assay and our intention to compare groups at the recovery time point, we justified the inclusion of the whole sample (all 18 participants) in the present analyses.

### Experimental design

**Fig. 1:**
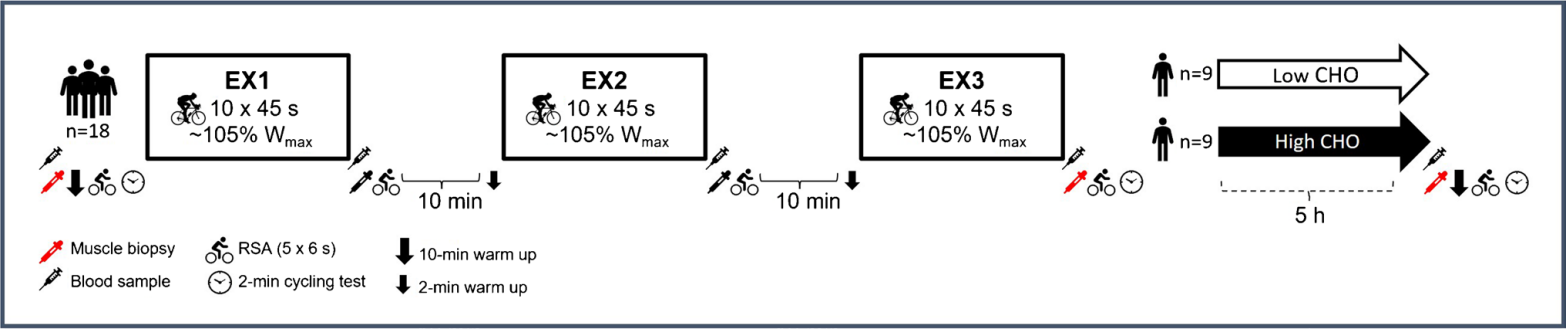
Overview of the main experimental day with muscle sampling (biopsy symbols highlighted in red denoting the samples used for maximal Na^+^-K^+^-ATPase activity measurements), blood sampling, repeated sprint ability (RSA) and the 2-min cycling test at standardized intensity for measurements of K^+^ release before and subsequent to three periods (EX1-EX3) of high-intensity intermittent exercise (10 × 45-s bouts with 135 s recovery) followed by randomization to low or high carbohydrate (CHO) fluid supplementation

The experimental setup has been described previously and is outlined in Fig. 1 [49, 50]. In short, following prior familiarization, the participants underwent a repeated high-intensity cycling session consisting of three periods of intermittent exercise (EX1-EX3) with muscle biopsies immediately before and after exercise, as well as after a 5-h recovery period with or without carbohydrate supplementation. An additional biopsy, not utilized for analyses in the present manuscript, was obtained after EX1. Repeated sprint testing was performed at baseline, after each period of intermittent exercise and at the recovery time-point to assess performance deteriorations and subsequent recovery, while a 2-min test at a standardized high intensity was conducted before and after the session, as well as at the recovery time point to assess K^+^ release during work-matched conditions. Blood was sampled frequently, and heart rate measured continuously during exercise. Participants were instructed to refrain from strenuous exercise 48 h prior to the experimental day, and for the last 24 h to refrain from tobacco, alcohol and caffeine.

### Main experimental day

On the experimental day, the participants arrived at the laboratory in the morning (8:00–9:00 am) after consuming a standardized breakfast ~ 1.5 h prior to arrival (~ 1.8 g carbohydrate·kg^−1^ bm through oats, skimmed milk, sugar, raisins, juice and water, amounting to ~ 22 kJ·kg^−1^ bm). After a 30-min rest period, the baseline muscle biopsy was obtained from the vastus lateralis muscle under local anesthesia and a blood sample obtained from an antecubital vein. Participants then completed a ~ 10-min warm up on a cycling ergometer before performing a baseline repeated sprint ability test (all tests are described in detail in subsequent sections). Next, a 5-min rest period was allowed before commencing 2 min of cycling at a standardized intensity of ~ 90% W_max_, as well as a limited number of maximal voluntary and electrically stimulated knee-extensor contractions (results presented elsewhere) [49]. After these baseline measurements, the main session comprising three periods of high-intensity intermittent cycling was initiated as described in detail below with repeated sprint testing and blood sampling performed after each of three exercise periods (Fig. 1). In addition, all assessments were repeated after completion of the three periods of high-intensity exercise including the post-exercise muscle biopsy obtained within ~ 30 s after cessation of exercise, a repeated sprint test, the 2 min of standard-intensity cycling and the knee-extensor contraction assessments. Thereafter, the participants rested for 5 h randomized to either a carbohydrate (CHO) or placebo (PLA) condition before a final muscle biopsy was obtained at rest and the same performance assessments as completed pre- and post-exercise were repeated at this recovery time point.

### High-intensity intermittent exercise protocol

A high-intensity intermittent exercise protocol was conducted on a Monark 894E Peakbike (Monark Exercise AB, Vansbro, Sweden). The protocol was chosen to provide a significant stimuli to induce deteriorations in sprint performance and potentially alter Na^+^-K^+^-ATPase activity, while at the same time reflecting the erratic activity pattern of intermittent sports. In addition, this protocol was expected to lead to significant glycogen depletion, enabling a clear separation between CHO and PLA supplemented groups during the recovery phase. The protocol consisted of three periods of 10 × 45-s cycling at ~ 105% W_max_ with each period separated by ~ 15 min of passive rest followed by a brief (~ 2 min) re-warm up before the next exercise period. The intensity of cycling was based on percentages of watt max (W_max_) obtained during incremental cycling (10 min at 120 W followed by 25 W increments each minute until exhaustion with a fixed cadence of 80 RPM) during the prior screening with minor adjustments incorporated following the familiarization visits to ensure completion of the protocol without premature exhaustion. In each 45-s bout, the individual resistance was fixed and sudden changes in cadence (and hence work rate) implemented in a systematic pattern as follows: 10 s at 108 RPM (~ 120% W_max_), 10 s at 90 RPM (~ 100% W_max_), 15 s at 101 RPM (~ 112% W_max_) and 10 s at 90 RPM (~ 100% W_max_). The cadence was controlled by live feedback on a screen in front of the participants through the Monark Anaerobic Test Software (Version 3.3.0.0) where data was sampled at a 1 Hz frequency and processed. Constant supervision and feedback were provided by the researchers to guide the cadence shifts. Repeated sprint ability was assessed as the average performance of the five sprints in a repeated sprint series. For each of these five sprints, the 3-s period with the highest average power was extracted and used to calculate the average of the test to avoid potential variation from participants failing to start at the exact time or from brief fluctuations in power readings.

### Repeated sprint ability

The warm up and subsequent repeated sprint ability test was performed on an electronically braked cycle ergometer (Schoberer Rad Messtechnik (SRM), 117 GmbH, Germany) before the high-intensity exercise session, after each of EX1-EX3 and at the recovery time point. This was incorporated to enable direct assessments of repeated sprint performance and evaluate the association between changes in performance and physiological read-outs (such as Na^+^-K^+^-ATPase activity). This consisted of five 6-s maximal sprints separated by 24-s rest periods to assess performance as previously described [50]. Data were sampled at 3 Hz and processed in SRM software (version 6.41.04).

### 2-min cycling test at a standardized high intensity

The 2-min cycling test at a standardized high intensity test was performed at the Monark cycling ergometer at an exercise intensity corresponding to ~ 90% Wmax (constant cadence of 80 RPM) to measure K^+^ accumulation in venous blood during work-matched conditions pre and post-exercise, as well as at the recovery time point. This intensity was chosen to select the maximal intensity which we would expect the participants to be able to maintain for 2 min both in a fresh and fatigued state. Of note, the measured blood potassium values significantly underestimate actual interstitial levels, however, this test was incorporated to detect potential differences between time points and/or groups. A blood sample was drawn just before and immediately after cessation of exercise. It should be noted that the recovery of K^+^ occurs rapidly (within the first minute of exercise cessation) meaning that small variations in sampling time are significant. Samples in the present project were obtained as rapidly as possible within 10–30 s post-exercise (the upper limit reflecting some delays in sampling).

### Carbohydrate and placebo supplementation

After completion of the high-intensity exercise protocol and associated performance assessments, carbohydrate or placebo supplementation was provided for the participants randomized to CHO or PLA during the 5-h recovery period. The CHO supplementation consisted of liquids and energy bars (Maxim; Orklacare A/S, Ishøj, Denmark) yielding a total of ~ 5.2 g CHO·kg^−1^ (~ 1 g CHO·kg^−1^·h^−1^). The PLA supplementation consisted of equal amounts of coloured and sweetened sugar free liquid (sugar free orange juice, Budget; Salling Group, Brabrand, Denmark). A small proportion of low-carbohydrate energy bars were also provided (soft bar; EASIS A/S, Midtjylland, Denmark) yielding a total of 0.3 g CHO·kg^−1^. The amounts of fat and protein were similar in each group (0.3 g·kg^−1^ of fat and protein). The supplementation was administered in four equal-sized portions ingested immediately following exercise and 45 min, 2 h and 3.5 h into recovery. The intervention was double-blinded since one staff member handling the supplementation was uninvolved in all other aspects of the study procedures, while the participants were unaware of the allocated condition (blinding was deemed successful as 50% guessed the correct allocation, but of these only half specified that they were somewhat certain or very certain of the allocated condition).

### Muscle sampling and analyses

Muscle biopsies were obtained from the right leg pre-exercise and from the left leg post-exercise and from the right leg again at the recovery time point. An additional sample not used in the present part of the analyses was obtained after the first exercise period from the left leg. The samples on the same leg were separated by ~ 3 cm with the latter biopsy taken more proximally. The muscle samples (150–200 mg w.w.) were divided into smaller portions for distinct analyses with the analyses included in the present manuscript including muscle glycogen content, metabolites, maximal Na^+^-K^+^-ATPase activity and Na^+^-K^+^-ATPase isoform expression measured from a large piece instantly frozen in liquid nitrogen. Approximately one third of the piece (~ 50 mg w.w) was stored at -80° C for later maximal Na^+^-K^+^-ATPase activity measures whereas the remaining part was freeze-dried and dissected in smaller portions, while being stored at -80° C before and after handling.

### Measurements of maximal in vitro Na^+^-K^+^-ATPase activity

**Fig. 2:**
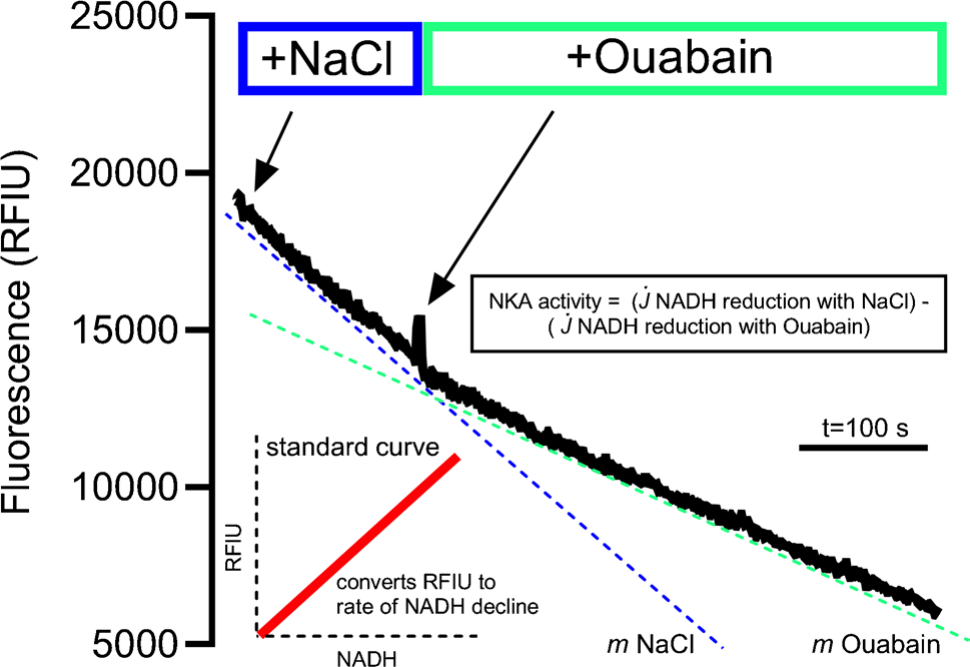
Representative trace and overview of the maximal Na^+^-K^+^-ATPase activity assay

Maximal in vitro Na^+^-K^+^-ATPase activity was measured using an NADH coupled assay as described in detail by Jannas-Vela et al. [46] with minor modifications. In short, the method measures Na^+^-K^+^-ATPase activity indirectly through an enzyme-linked approach with an ATP regenerating assay facilitated by glycolytic reactions (pyruvate kinase and lactate dehydrogenase steps) to couple ATP turnover to NADH degradation. The NADH degradation rate is measured fluorometrically (Lumina; Thermo Scientific, Fisher, Hampton, NH) at an excitation wavelength of 340 nm and an emission wavelength of 460 nm with the change in slope before and after Na^+^-K^+^-ATPase inhibition with ouabain used to calculate maximal Na^+^-K^+^-ATPase activity (maximal Na^+^-K^+^-ATPase dependent ATP hydrolysis), see Fig. 2 for representative trace.

Before performing the assay, muscle samples were diluted (11:1 vol/wt) in ice-cold buffer (0.2 mM PMSF, 250 mM sucrose, 5 mM HEPES and 0.2% NaN3 (pH 7.5) and homogenized using a handheld glass mortar and pestle for separation into multiple aliquots. A Bradford assay was performed to determine protein content of the homogenate before aliquots were snap-frozen and stored at -80° C until further processing. When initiating the assay, muscle aliquots were first freeze-thawed 5 times in liquid nitrogen while a cuvette containing ~ 1.5 mL of reaction buffer (50 mM Tris–HCl, 15 mM KCl, 1 mM EGTA, 4 mM MgCl2, 2.5 mM PEP, 4 mM MgATP, and 5 mM KN3 (pH 7.4)) was prepared by heating for 10 min to 37 °C. Before starting the reaction, NADH (Sigma-Aldrich, St- Louis, MO) was added to saturating levels and 3.5 μL of pyruvate kinase (Sigma-Aldrich) and lactate-dehydrogenase (Sigma-Aldrich) were also added. The reaction was started by addition of 80 mM NaCl in the presence of 25 μl blebbistatin. The Na^+^-K^+^-ATPase dependent ATP hydrolysis was obtained by the addition of 2 mM ouabain to inhibit the Na^+^-K^+^-ATPase and the change in slope was used to calculate the Na^+^-K^+^-ATPase dependent ATP hydrolysis in relation to a standard curve previously determined for fluorescence intensity and NADH levels under the same assay conditions:

$$\mathrm{Na}^+\text{-}\mathrm K^+\text{-}\mathrm{ATPase}\;\mathrm{activity}\;\left(\mu\mathrm{molmin}^{-1}\mathrm g^{-1}\mathrm{protein}\right)=\left(\left(j_{\left[Naci\right]}-j_{\left[Ouab\right]}\right)/m/\mu\mathrm g\;\mathrm{of}\;\mathrm{protein}\right)\times1000$$

With j_[NaCl]_ being the change in NADH in the presence of Na^+^, and j_[Ouab]_ being the change in NADH after addition of 2 mM ouabain generated by converting the relative fluorescence intensity unit (RFIU) into NADH using a standard curve (*m* being the slope obtained from the NADH standard curve).

Samples were run in duplicate or triplicate. A few samples were non-responsive to ouabain inhibition and excluded from the analyses (this included 0 samples at baseline, 2 samples post-exercise and 2 samples at the recovery time point). Furthermore, one biopsy sample was missing for Na^+^-K^+^-ATPase activity measurements due to insufficient tissue yield at the recovery time point.

### Muscle glycogen and metabolites

Muscle glycogen was measured using a spectrophotometer (Beckman DU 650) as described in detail previously [51]. Muscle lactate, PCr and Cr were measured with freeze-dried muscle tissue (~ 10 mg dw), extracted with 0.5 M HClO_4_ and analyzed using enzymatic methods [52]. In brief, extracts were centrifuged for 30 s and the supernatants initially frozen before conducting the specific analyses. Metabolite concentrations were normalized to the total creatine content (PCr + Cr) at each respective time point to adjust for weighing variability and sample contamination.

### Myosin heavy chain composition

Fiber type composition was determined from homogenate using gel electrophoresis and quantified densitometrically, as previously described [53]. Muscle homogenate (20 µl) and sample buffer (100 µl, 10% glycerol, 5% 2-mercaptoethanol, 2.3% SDS, 62.5 mM tris, 0.2% bromophenolblue at pH 6.8) were mixed, boiled for 3 min in water and loaded on a SDS-PAGE gel (6% polyacrylmide (100:1 acrylmid:bis-acrylmid), 30% glycerol, 67.5 mM tris-base, 0.4% SDS and 0.1 M glycine) using three different protein quantities (25–40 μg). The gels were run at 4 °C at 80 V for a minimum of 42 h and MHC bands made visible by Coomassie staining. Fiber type was calculated from the average of one biopsy from each leg and from three separate lanes for each biopsy.

### Western blots of Na^+^-K^+^-ATPase isoforms

Western blots were performed using a piece of muscle from the resting muscle biopsy pre-exercise. However, due to insufficient tissue yield and based on the assumption of no change in protein content within the context of the present study, some of the analyses were performed using tissue from one of the subsequent biopsies (n = 8). The freeze-dried muscle samples were divided in two different tubes for each biopsy and homogenized in fresh cold homogenization buffer containing (in mM) (10% glycerol, 20 Na-pyrophosphate, 150 NaCl, 50 HEPES (pH 7.5), 1% NP-40, 20 β-glycerophosphate, 2 Na3VO4, 10 NaF, 2 PMSF, 1 EDTA (pH 8), 1 EGTA (pH 8), 10 µg/ml aprotinin, 10 µg/ml leupeptin, and 3 µg/ml benzamidine. The samples were subsequently homogenized in a tissuelyser (Qiagen Tissuelyser II, Retsch GmbH, Haan, Germany) for 2 × 2 min at 28.5 Hz. For 1 h after this, samples were rotated end over end at 4 °C, and thereafter sonicated (Branson Digital Sonifier) 1 × 10 s at 10% amplitude. The resulting supernatant was used for further analysis as described in detail previously [54]. The primary antibodies used in the study were optimized (for details; see [55]) and included: Na^+^-K^+^-ATPase β1 isoform: 40–45 kDa (MA3-930, Affinity Bioreagents); Na^+^-K^+^-ATPase α1 isoform: 100 kDa (α6F Developmental Studies Hybridoma Bank, Iowa, USA) and Na^+^-K^+^-ATPase α2 isoform: 100 kDa (07–674 Millipore). The secondary antibodies used were HRP conjugated goat anti-mouse, rabbit anti-goat (P-0447 and P-0449 DAKO, Denmark), and goat anti-rabbit (4010–05 Southern Biotech, Birmingham AL, USA).

### Blood sampling and K^+^ analyses

Blood was drawn in lithium-heparin and serum-tubes, centrifuged and subsequently separated in portions for distinct analyses. Lithium-heparin tubes were centrifuged immediately at 4 °C and plasma transferred to new tubes and stored at -20 °C. Plasma potassium concentration was measured using a flame photometer (Radiometer FLM3), with lithium as the internal standard.

### Statistical analyses

A linear mixed-effects model was used to determine changes in maximal Na^+^-K^+^-ATPase activity (or other dependent variables) with time as fixed effect and participant as random effect. Furthermore, an additional analysis was performed with group added as fixed effect and an interaction link between group and time incorporated to determine potential effects of the carbohydrate manipulation following the exercise session. Data normality and heteroscedasticity were assessed by inspection of the distribution of residuals and normal probability plots. Correlation coefficients were determined for Na^+^-K^+^-ATPase activity and performance/muscle variables using Pearson correlation analyses and interpreted as trivial (r = 0.1–0.29), moderate (r = 0.3–0.49), large (r = 0.5–0.69), very large (r = 0.7–0.89), nearly perfect (r = 0.9–0.99), and perfect (r = 1.0) [56]. Data are presented as means ± SD. Significance level was set at *P* ≤ 0.05. Statistical analyses were performed using Stata/IC16 (StataCorp, College Station, TX, USA) and figures created in GraphPad Prism Version 10.1.0 (GraphPad Software Inc., San Diego, California, USA).

## Results

### Work pattern and muscle metabolism

**Fig. 3:**
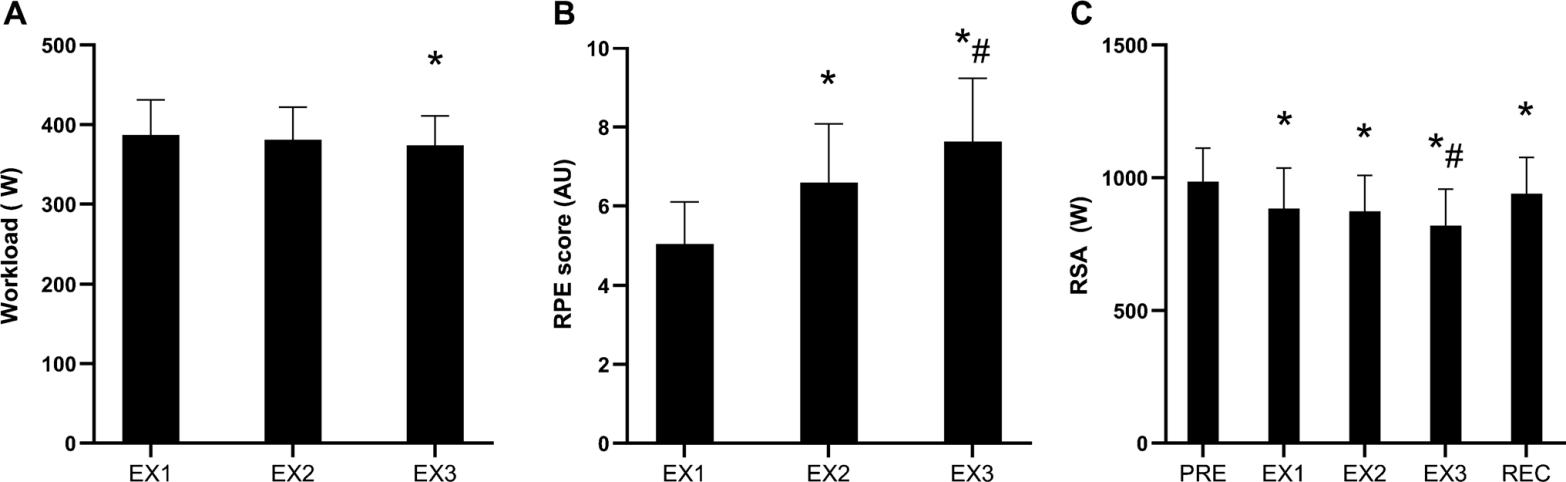
Average workload (**A**) and ratings of perceived exertion (**B**) during each of the three high-intensity exercise periods (EX1-EX3), as well as mean repeated sprint ability (RSA) pre-exercise, post EX1-EX3 and at the recovery time point (Rec) for the pooled sample (**C**). Data are presented as means ± SD, n = 18. * denotes significant difference from baseline/EX1; # denotes significant difference from EX1 and EX2; *P* ≤ 0.05

The general session characteristics including work performed and muscle metabolic responses have been presented previously [50] and key variables are therefore included only to characterize the exercise performed, see Fig. 3 for overview. The total work load completed was slightly lower during EX3 (*P* = 0.002) due to small reductions in resistance to allow completion of the full session and corresponded on average to 107 ± 5% W_max_ across the complete exercise session (Fig. 3a). Ratings of perceived exertion increased progressively reaching maximal or near-maximal levels at the point of exhaustion (*P* < 0.001). Repeated sprint ability dropped markedly by 17% after the complete exercise session (*P* < 0.001), and was only partially recovered after the recovery period with a persistent 5% reduction at this point for the pooled sample (*P* = 0.007; including participants both with and without carbohydrate supplementation). When separating repeated sprint ability by condition, this was recovered to baseline levels in the carbohydrate-supplemented group, while remaining slightly lowered by ~ 8% in the placebo condition (*P* < 0.001) as described herein [49].

**Fig. 4:**
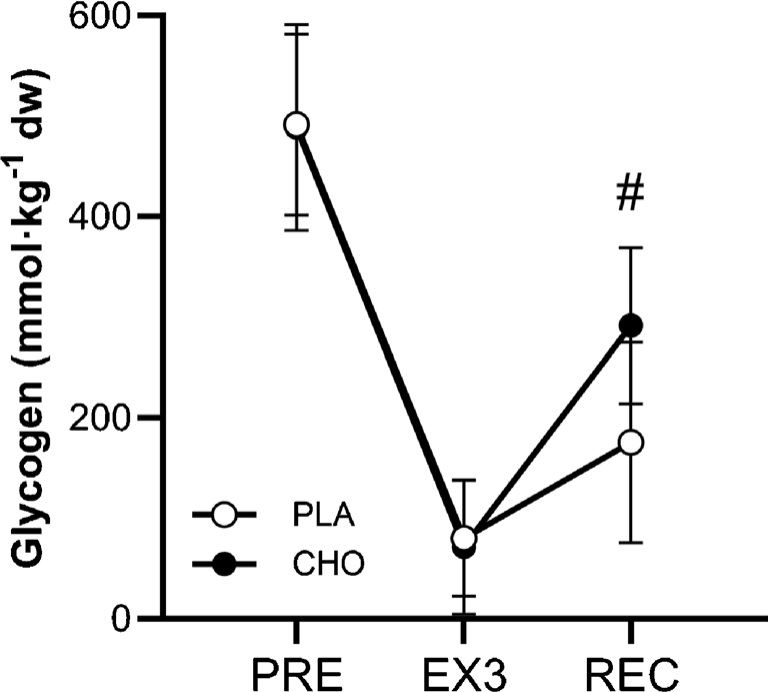
Muscle glycogen concentrations measured pre-exercise, post-exercise and after a 5-h recovery period with PLA and CHO supplementation. Data are presented as means ± SD, *n* = 18. # denotes significant between-group difference; *P* ≤ 0.05

Muscle glycogen declined from 490 ± 94 mmol·kg^−1^ dw at baseline to 76 ± 94 mmol·kg^−1^ dw post-exercise (*P* < 0.001) and was partly resynthesized at the recovery time point to 234 ± 105 mmol·kg^−1^ dw (*P* < 0.001), see Fig. 4. The glycogen levels were higher in the carbohydrate vs. placebo-supplemented group at this time point (291 ± 78 vs. 175 ± 100 mmol·kg^−1^ dw, *P* = 0.020 recovering from 72 ± 67 and 80 ± 58 mmol·kg^−1^ dw in CHO and PLA, respectively). Muscle lactate values rose from 9.7 ± 3.7 mmol·kg^−1^ dw at baseline to 43.1 ± 24.0 mmol·kg^−1^ dw after the complete exercise session (*P* < 0.001).

### Exercise-induced changes in maximal Na^+^-K^+^-ATPase activity

**Fig. 5:**
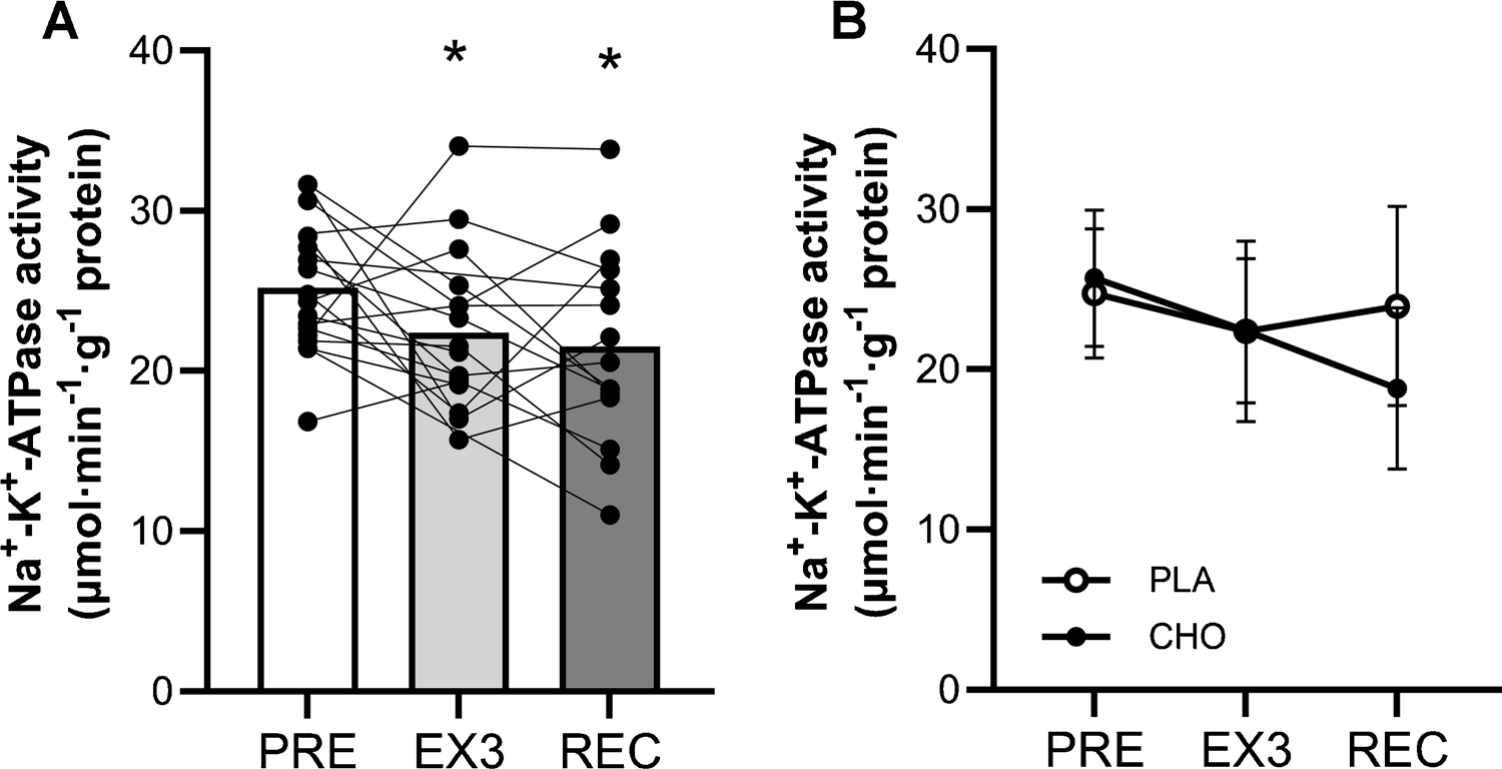
Maximal in vitro Na^+^-K^+^-ATPase activity measured pre-exercise, post-exercise and after a 5-h recovery period for **A**) pooled data for carbohydrate and placebo groups with individual values and **B**) data summarized for the carbohydrate and placebo groups. Data are presented as means ± SD, *n* = 15–18. * denotes significant difference from baseline; *P* ≤ 0.05

The complete exercise session resulted in a significant 12 ± 6% reduction in maximal Na^+^-K^+^-ATPase activity (Fig. 5a, *P*= 0.039). This decline was sustained after a 5-h recovery period when pooled data for participants with/without carbohydrate intake was assessed (15 ± 6% decline compared to baseline, *P* = 0.008). When including an interaction link to assess potential effects of the carbohydrate manipulation, no significant time x group interaction was observed, although a tendency (*P* = 0.078) was present for a decline in absolute activity in the carbohydrate supplemented compared to the placebo group (-4.9 ± 2.8 μmol·min^−1^·g^−1^ protein, see Fig. 5b).

### Plasma potassium

**Fig. 6:**
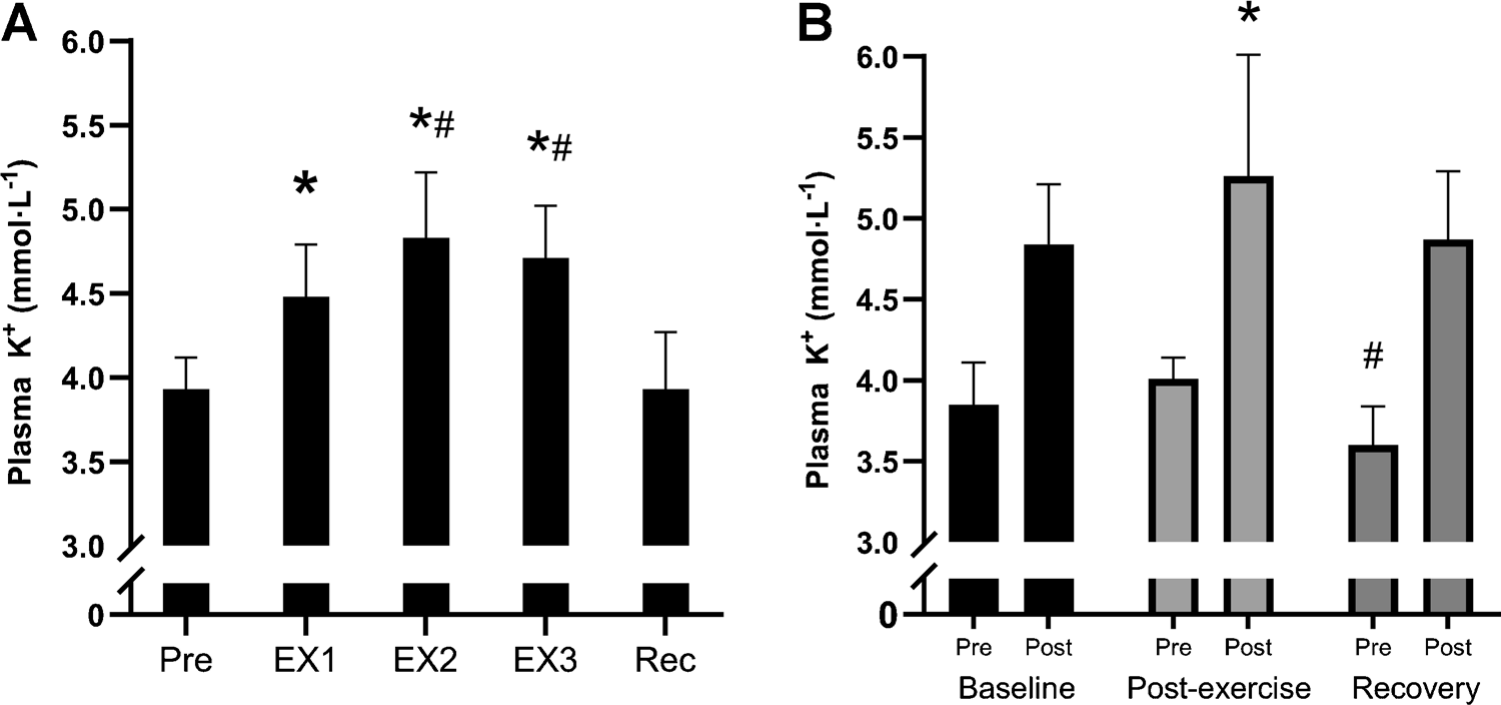
**A**) Plasma K^+^ concentration at baseline (Pre), after each period of high-intensity intermittent exercise (EX1-EX3), as well as after 5 h of recovery (Rec); and **B**) plasma K^+^ levels before (Pre) and after (Post) the 2-min cycling test at a standardized high intensity performed at baseline, post-exercise and after recovery (Rec). Data are presented as means ± SD. * denotes significant difference from baseline and recovery in (A) and for corresponding post values in (B); # denotes significant difference from EX1 in (A) and significant difference from corresponding pre values in (B); *P* ≤ 0.05

Plasma potassium increased during the high-intensity intermittent exercise session from 3.9 ± 0.2 mmol·L^−1^ at baseline to 4.5 ± 0.3 mmol·L^−1^ post EX1 (Fig. 6a, *P* < 0.001). This level increased further to 4.8 ± 0.4 mmol·L^−1^ after EX2 (*P* < 0.001) and was sustained elevated at this level after EX3 (4.7 ± 0.3 mmol·L^−1^, *P* = 0.018 compared to after EX1 and *P* = 0.223 compared to after EX2) despite the reduced workload at this time point. After the 5-h recovery period, plasma K^+^ returned to baseline levels of 3.9 ± 0.3 mmol·L^−1^ (*P* = 0.952).

The plasma K^+^ levels rose by ~ 25% during the pre-exercise 2-min test (*P* < 0.001), however, this increase tended to be greater after the post-exercise 2-min test (31% increase, *P* = 0.103) and after that at the recovery time point (35% increase, *P* = 0.062) despite the work-matched conditions (Fig. 6b). The absolute pre-test values were 3.85, 4.01 and 3.6 mmol·L^−1^ after the 2-min test at baseline, post-exercise and at the recovery time point with the level at the recovery time point being significantly lower than at baseline (*P* = 0.015) and post-exercise (*P* < 0.001), with no differences between baseline and post-exercise (*P* = 0.220). Post-test levels rose to 4.8, 5.3 and 4.9 mmol·L^−1^ at baseline, post-exercise and recovery, with the post-test level after the high-intensity exercise session being higher than after the test at baseline (*P* < 0.001) and after the test at the recovery time point (*P* = 0.001). No difference was present for the post-test value at the recovery time point compared to the post-test value at baseline (*P* = 0.946).

### Relationships between maximal Na^+^-K^+^-ATPase activity, muscle protein expression and exercise performance at baseline, fatigue and recovery

**Fig. 7:**
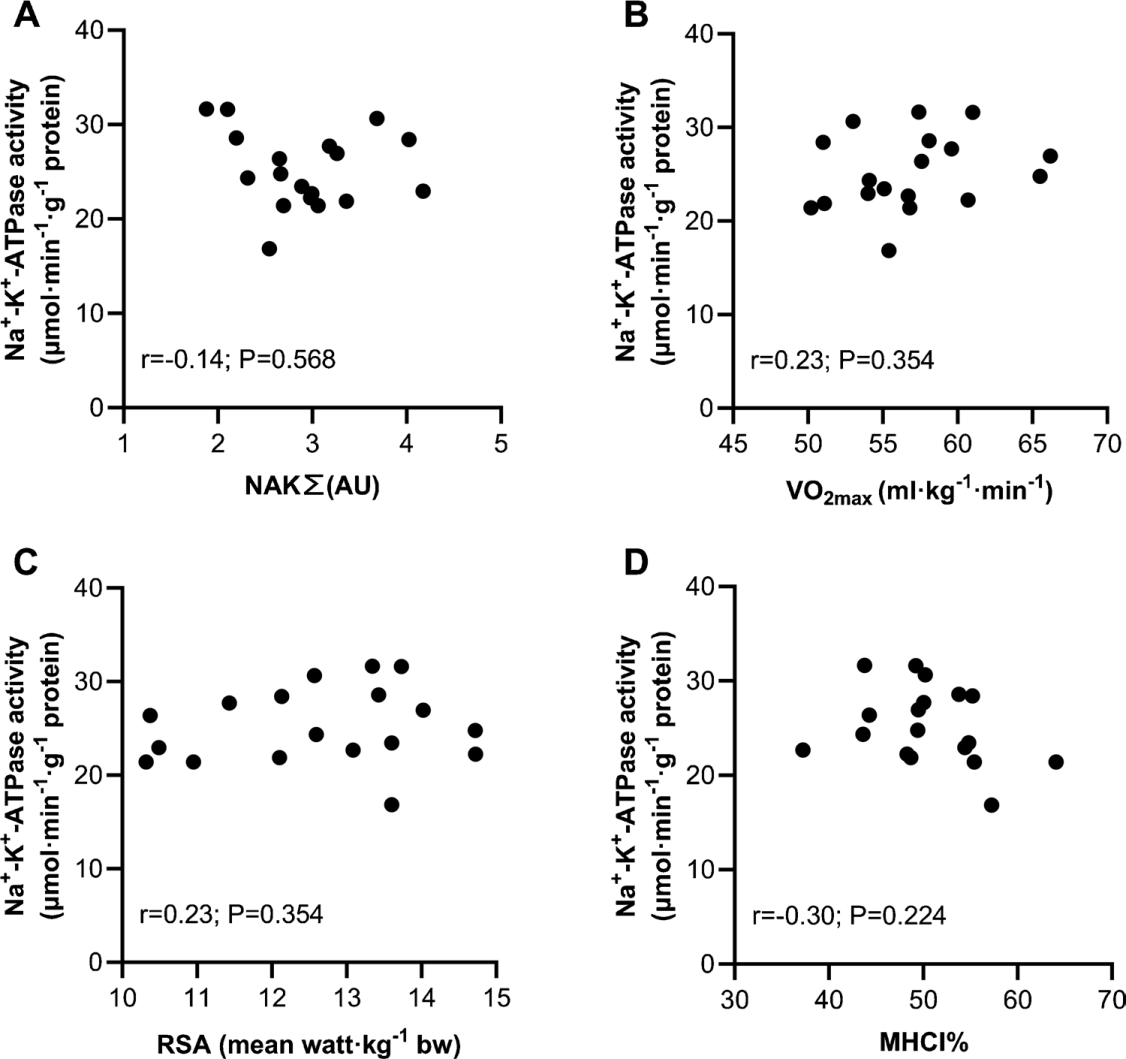
Baseline relationships between maximal in vitro Na^+^-K^+^-ATPase activity and participant characteristics. **A**) Na^+^-K^+^-ATPase activity and the sum of Na^+^-K^+^-ATPase subunit isoforms α1, α2 and β_1_, **B**) Na^+^-K^+^-ATPase activity and VO_2max_, **C**) Na^+^-K^+^-ATPase activity and repeated sprint ability (mean of 5 sprints relative to body mass) and **D**) Na^+^-K^+^-ATPase activity and % myosin heavy chain 1 (MHC). *n *= 18; *P* ≤ 0.05

The maximal Na^+^-K^+^-ATPase activity at baseline was unrelated to the expression of Na^+^-K^+^-ATPase subunits (Fig. 7a, sum of all isoforms; Na^+^-K^+^-ATPase β1, α1 and α2) This was also the case when performing the correlation analysis with inclusion only of Western Blots performed on the same piece of muscle as the activity assay (r = -0.47, *P* = 0.568). Furthermore, no significant relationships were present for baseline maximal Na^+^-K^+^-ATPase activity and VO_2max_ or mean repeated sprint ability expressed relative to body mass (Fig. 7b and c), as was the case for the fatigue index (actual watt output as a total of all 5 sprints compared to the ideal output if the fastest single sprint was maintained in each repetition) obtained in the repeated sprint test (r = 0.25, *P* = 0.320). Finally, no significant relationship was found with fiber type composition (Fig. 7d).

**Fig. 8:**
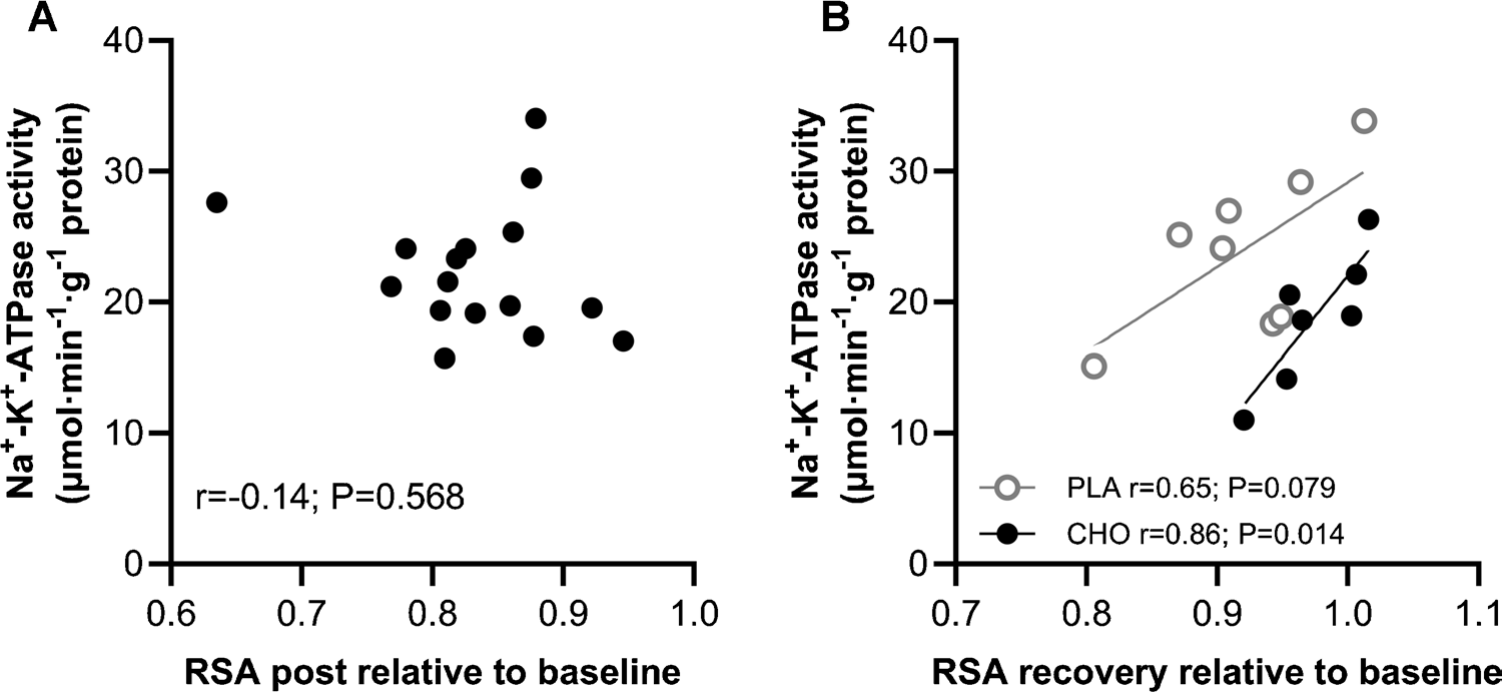
Maximal in vitro Na^+^-K^+^-ATPase activity post-exercise (**A**) and at the recovery time point (**B**) in relation to the repeated sprint ability at the corresponding time point normalized to baseline performance. *n* = 15–16; *P* ≤ 0.05

The exercise-induced decline in repeated sprint ability measured relative to the baseline level was not associated with the absolute Na^+^-K^+^-ATPase activity when assessed immediately post-exercise (r = -0.14, *P* = 0.568). When measured at the recovery time point, no association was present for the pooled sample (r = 0.27, *P* = 0.333). However, when the CHO and PLA groups were analyzed independently, a strong significant relationship was present in the CHO group (r = 0.86, *P* = 0.014), while repeated sprint ability relative to baseline tended to correlate with the absolute Na^+^-K^+^-ATPase activity in the PLA group (r = 0.65, *P* = 0.079) (Fig. 8a and b).

## Discussion

The key finding of the present investigation was a significant (12%) reduction in maximal Na^+^-K^+^-ATPase activity after a high-intensity intermittent exercise session demonstrated using a novel NADH-coupled ATP regenerating assay. Interestingly, this impairment was sustained after a 5-h recovery period and was not affected by carbohydrate intake and subsequent distinctions in muscle glycogen concentrations under the present experimental/analytical conditions.

A reduction in maximal Na^+^-K^+^-ATPase activity of this magnitude is in line with the majority of previous studies applying various exercise modalities. For example, reductions have been found following prolonged, moderate-intensity exercise sessions [24, 26, 27, 29, 40], isolated knee-extensor muscle contraction protocols [21, 22, 28, 37] and single bouts of continuous or incremental intense exercise of short duration [18, 19, 23, 25, 30]. Only one previous study [19] adopted an intense intermittent exercise protocol, comprising eight 5-min intervals at ~ 85% VO_2max_, yielding a comparable ~ 13% decline in maximal enzyme activity (maximal 3-O-MFPase activity) as in the present study.

Importantly, contrary to previous investigations, we adopted a different methodological approach to assess maximal in vitro Na^+^-K^+^-ATPase activity taking advantage of a specific myosin ATPase inhibitor to substantially reduce background noise and yield maximal enzyme activities three-to-four times higher than previously reported [46]. In addition, unlike the most common assay in the literature (the 3-O-MFPase activity assay), the present assay is Na^+^-sensitive, which is a major asset since Na^+^ is a key regulator of the Na^+^-K^+^-ATPase. Thus, by adopting the present methodological approach, we support previous investigations delineating a reduction in maximal Na^+^-K^+^-ATPase activity as a potential feature of muscle fatigue.

The observed reduction in maximal Na^+^-K^+^-ATPase activity is unlikely to reflect a loss of pumps since previous studies have found no change in ouabain binding site content (as a measure of total pump concentration) or single isoform protein abundance (Western blotting) following brief, prolonged or intermittent exercise sessions [18, 20, 21, 26]. It is more likely that the reduction in enzyme activity is related to a modification of the existing pumps, with prevailing mechanisms including inhibitory actions of reactive oxygen species and oxidative modifications involving the formation of disulphide bonds between glutathione and reactive cysteine thiols (S-glutathionylation) [57]. Experiments in both exercising humans and electrically-stimulated isolated rat skeletal muscle reveal an increase in the glutathionylation of Na^+^-K^+^-ATPase isoforms with muscle contractions in association with decreased maximal enzyme activity [35, 36]. Moreover, a dose-dependent reduction in maximal Na^+^-K^+^-ATPase activity was reported following in vitro glutathionylation of human skeletal muscle [35] possibly due to the blocking of the ATP binding site by glutathione [58]. Vice versa, an increase in maximal Na^+^-K^+^-ATPase activity has been reported when treating rat skeletal muscle with the reducing agent dithiothreitol (DTT) [34], while intravenous N-acetylcysteine infusion (antioxidant) attenuates the exercise-induced reduction of maximal pump activity in humans [27], supporting an important role of reactive oxygen species.

Interestingly, the depressing effect of exercise on maximal Na^+^-K^+^-ATPase activity was sustained after a 5-h recovery period in the presence or absence of carbohydrate intake and with no beneficial effect of carbohydrate ingestion (in fact a trend toward a negative effect was observed, probably attributable to a type 2 error). Consequently, this reduction is unlikely to be directly related to muscle glycogen concentrations, since maximal enzyme activity remained attenuated after a 5-h recovery period independent of the degree of restoration of glycogen levels. This is in opposition to findings in intact rodent muscle or skinned single fibers where muscle glycogen concentrations and glycolytic ATP production have been closely linked with changes in muscle excitability, thought, at least in part, to reflect changes in maximal Na^+^-K^+^-ATPase activity [15, 32, 59]. For example, reductions in action potential repriming period (indicative of impaired Na^+^-K^+^-ATPase activity) was observed in skinned fibers with chemical inhibition of glycogenolysis, despite the bathing solution being rich in PCr and ATP [32]. Moreover, glycolytic enzymes are situated in the narrow triadic junctions between the T-tubular system and sarcoplasmic reticulum where bulk ATP has been shown not to be in equilibrium [60]. Instead, glycolytic intermediates have been suggested to channel into these junctional gaps and in that way constitute a critical substrate for local ATP regeneration [60].

Studies in exercising humans delineating the role of glycogen concentration on muscle excitability and Na^+^-K^+^-ATPase function are lacking. However, it has been demonstrated that severe glycogen depletion in a proportion of muscle fibers and/or subcellular glycogen storage compartments manifests at whole-muscle glycogen levels below ~ 250 mmol·kg^−1^ dw [50]. Accordingly, this level of glycogen depletion has been proposed as a potential threshold, below which muscle function and excitation–contraction coupling processes may be affected [61, 62]. Thus, the level of glycogen depletion induced post-exercise in the present study (~ 75 mmol·kg^−1^ dw) was well below this proposed critical level. Similarly, at the recovery time point, a relatively low glycogen level was sustained in the placebo group (171 mmol·kg^−1^ dw), while recovery to levels marginally above the proposed threshold was obtained in the carbohydrate group (291 mmol·kg^−1^ dw). Therefore, we cannot rule out the possibility that larger distinctions in glycogen levels would yield different results. Furthermore, it has previously been shown, using the 3-O-MFPase activity assay, that glucose ingestion during exercise resulted in increases in maximal Na^+^-K^+^-ATPase activity by mechanisms that are unclear [40]. Yet, it is not surprising that potential metabolic perturbations were not captured in the present setting under the applied optimal assay conditions (e.g. sustained high ATP concentrations).

The lack of recovery of pump function during the time frame of the present study is in contrast to results obtained by Fowles et al. [21], where full recovery was observed after a 4-h rest period following a 30-min isometric knee-extensor exercise session, having induced a 35% reduction in pump activity post-exercise. However, only 9 participants were biopsied in the study, likely resulting in inadequate power to confidently assess changes given the inherent assay variability. Indeed, only a strong trend toward an impairment in maximal Na^+^-K^+^-ATPase activity was found post-exercise in the exercised leg compared to the pre value while the comparison with a non-exercised control leg yielded the significant finding reported. Moreover, Petersen et al. [28] reported an 11% reduction in maximal pump activity following a brief (~ 6 min) dynamic knee-extensor fatigue protocol, which was reversible by 3 h of recovery, possibly explained by the markedly lower amount of total work than in the present study. In support of our results, Green et al. [23] observed an incomplete recovery of maximal Na^+^-K^+^-ATPase activity between repeated 6-min bouts of intense exercise (~ 91% VO_2max_) interspersed by 54 min of recovery every hour for 16 h in a row, resulting in a progressive decline in pump function. While the exact time-course of impairments and subsequent recovery of maximal Na^+^-K^+^-ATPase activity is not well characterized, these results suggest that the observed reductions reflect a long-lasting downregulation rather than acute alterations, perhaps dependent on the amount of work performed.

An enticing question is then to what degree maximal Na^+^-K^+^-ATPase activity assessed in vitro reflects in vivo capacity*;* and, to what degree these alterations affect muscle fatigue and performance. To some degree, a dissociation between the in vitro and in vivo conditions is expected due to the apparent insensitivity to declines in muscle glycogen levels in addition to potential localized perturbations in phosphocreatine and ATP during muscle contractions since the assay is performed under presumably optimal conditions (e.g. high ATP levels) [63]. Hormones such as insulin or catecholamines additionally modulate enzyme activity by increasing Na^+^-K^+^-ATPase affinity for Na^+^ in contracting muscle [64]. Adding to the complexity, there is mounting evidence from rodent models [65–67] suggesting that moderate perturbations of extracellular K^+^ may potentiate muscle force production. Consequently, excitation-induced flux of K^+^ to the interstitium may exert a dual role in regulating muscle force production during exercise through depression or potentiation, depending on the specific setting [8]. Furthermore, exacerbated escalations in K^+^ levels accompanying a decreased pump activity, leading to reduced muscle cell excitability, may be rescued by concomitant changes in ClC-1 opening state (e.g. through reduced pH) [10, 68]. However, with repetitive action potential firing and/or metabolic perturbations (ATP depletion or reduced glycolytic rate), ClC-1 and K_ATP_ channels simultaneously open and markedly limit cell excitability [10, 69]. In this way, regulation of ClC-1 and K_ATP_ opening state are also prime candidates for a coupling between reduced muscle glycogen levels and impaired cell excitability in addition to modulation of the Na^+^-K^+^-ATPase.

In the present study, no correlations were present at baseline between maximal Na^+^-K^+^-ATPase activity and repeated sprint performance. Furthermore, no correlation was observed between the decline in repeated sprint ability immediately (~ 2 min after) post-exercise and the reduction in maximal enzyme activity. However, at this point, clearly a multitude of acute fatigue mechanisms interact to determine performance responses and a lack of significance does not rule out a potential role of altered Na^+^-K^+^-ATPase activity. Moreover, an increase in absolute plasma K^+^ levels was observed at this time point in addition to a trend (P = 0.103) toward a larger increase in K^+^ during the work-matched 2-min test. To rule out some of these factors, we studied the 5 h recovery time-point where the severe acute metabolic perturbations affecting muscle function are no longer expected to be present. This revealed a positive relationship between repeated sprint ability (relative to the baseline level) and absolute Na^+^-K^+^-ATPase activity but without reaching statistical significance. Strong positive associations were present though, when distinguishing between the placebo and carbohydrate supplementation groups (in which repeated sprint ability was independently affected by the muscle glycogen manipulation [49]) suggesting a potential role for the reduction in maximal Na^+^-K^+^-ATPase activity in the impaired repeated sprint ability at this point. In addition, the increase in K^+^-levels during the 2-min test again approached statistical significance (P = 0.062) in the direction of a larger increase compared to the baseline test, potentially reflecting an impaired ability of the Na^+^-K^+^-ATPase to regulate ion homeostasis.

A relationship between maximal pump activity and performance has, to the best of our knowledge not been routinely assessed in the existing literature. The study by Fowles et al. [21] which reported a restoration of maximal Na^+^-K^+^-ATPase activity after a 4-h recovery period simultaneously observed a persisting reduction in muscle function, suggesting a dissociation between measures of maximal Na^+^-K^+^-ATPase activity and performance. On the other hand, a study in hemodialysis patients showed a baseline reduction in maximal pump activity (~ 31%) compared to healthy controls, concomitant with a decrease in exercise performance despite no difference in ouabain binding sites and proposed that the impaired performance could be partly associated with reduced maximal pump activity [70]. Accordingly, in that study, a moderate (r = 0.45, P = 0.02) correlation was found for maximal Na^+^-K^+^-ATPase activity (3-O-MFPase activity) and VO_2peak._ This is both supported [22] and opposed [26] by the previous literature, and the findings observed herein delineate no association at baseline between maximal pump activity and VO_2max_. Thus, taken together, a relationship between maximal Na^+^-K^+^-ATPase activity and different types of exercise performance remains unclear.

In the present study, no association was observed between maximal Na^+^-K^+^-ATPase activity and our measure of Na^+^-K^+^-ATPase abundance through western blotting, which is consistent with studies by Leppik et al. [26] and Murphy et al. [71] (both using ouabain binding sites) but inconsistent with two other investigations [21, 22] measuring ouabain binding sites and maximal Na^+^-K^+^-ATPase activity (3-O-MFPase). However, it may well be that the variability in both the activity assay and the (semi) quantification of protein abundance using Western blotting precludes the ability to confidently assess correlations within the current sample size.

Finally, our absolute Na^+^-K^+^-ATPase activity results are somewhat higher than the proposed theoretical maximal values suggested by Jannas-Vela et al. [46]. This may be explained by the higher training status of the present sample (young moderately-to-well trained participants vs. healthy older adults) and hence a higher Na^+^-K^+^-ATPase content which is the basis for the quantification of the theoretical maximal level. Thus, our results reflect maximal values expected from the present study cohort.

## Conclusion

In conclusion, our results support an exercise-induced depression of maximal Na^+^-K^+^-ATPase activity, using a novel methodological approach. This depression persisted after a 5-h recovery period, regardless of carbohydrate ingestion, likely reflecting structural modifications of the enzyme. In addition, maximal Na^+^-K^+^-ATPase activity was associated with the decline in repeated sprint ability at the recovery time point, though not immediately post-exercise, suggesting a potential, but yet not clear role in muscle fatigue.

## Data Availability

Data supporting the findings of the present study is available from the corresponding author upon reasonable request.
